# At 75 Years, Is Lithium the Gold Standard in Bipolar Disorder?

**DOI:** 10.3390/ph18121850

**Published:** 2025-12-04

**Authors:** Robert H. Belmaker

**Affiliations:** Department of Psychiatry, Faculty of Health Sciences, Ben Gurion University of the Negev, Beersheva 8410501, Israel; belmaker@bgu.ac.il

**Keywords:** lithium, bipolar disorder, gold standard, lithium response, mechanism of action, second-generation antipsychotics, antiepileptics

## Abstract

Lithium was the first discovered mood stabilizer for bipolar disorder. However, several antiepileptics such as valproate and carbamazepine have also proven to be mood stabilizers in all phases of bipolar disorder with profiles barely distinguishable from lithium. Second-generation antipsychotics were slowly recognized in almost all cases to be useful in all phases of bipolar disorder. These different treatments have not been found to have any common mechanism of action. Indeed, lithium itself has numerous biochemical effects that are often not replicable. The newest biochemical effects described for lithium are exciting but are still merely promising and should not be taught to medical students as facts, especially since they do not explain the fact that several antiepileptic and second-generation antipsychotics also work well in all phases of bipolar disorder. Lithium is not specific to bipolar disorder but has uses in several other psychiatric and nonpsychiatric conditions. Lithium, like most treatments in medicine, is nonspecific; it is still useful in many cases, but is not in any sense a “gold standard”.

## 1. Introduction

75 years since John Cade‘s foundational work on lithium in psychiatry is a proper time for celebration, reflection and reconsideration. Indeed, all of psychopharmacology is going through such a historical self-evaluation. Ed Shorter’s recent book *The Rise and Fall of the Age of Psychopharmacology* [1] describes the difficulties in industry-sponsored pharmaceutical company trials and the disappointment in the lack of progress in new antipsychotic and new antidepressant compounds. In the lithium area, Tom Ban’s scholarly *History of Lithium Research* [2] devotes great effort to ascribing credit for various steps along the way to lithium’s clinical use today and less on the actual current place of lithium in a psychopharmacological algorithm. Walter Brown’s best-selling book *Lithium: A Doctor, a Drug and a Breakthrough* [3] novelized the story of John Cade and made him into a hero without critical analysis of the fact that Cade himself refused to use lithium for most of his professional life after his early discoveries. Some recent journal articles have celebrated lithium as the “first choice” or “gold standard” for treatment of bipolar disorder [4]. However, other articles have emphasized the increasing concerns of long-term lithium treatment on the kidney [5,6] and on the parathyroid gland, even more than on the thyroid. Many articles have noted that in every major country where lithium has been widely used, lithium’s use is declining both on a per capita basis and as a percentage of total psychopharmaceutical medications sold. For some researchers, this is seen as a paradox: Why should a “gold-standard” treatment be facing declining use? However, such phenomena are the rule rather than the exception in medicine. The great impact of the first successful human heart transplant by Christian Bernard in 1967 has not led to increasing rates of heart transplants throughout the world and certainly no one would consider that good medicine implies that heart transplants should be on the rise every year. The impact of Christian Bernard’s successful heart transplant was the fact that it made waves in transplant research in many other disciplines, stimulated the development of medicines to combat transplant rejection, and inspired continuing work in the development of non-biological artificial hearts. Similarly, the discovery of the first antibiotic sulfa drug was of huge importance but no one would measure that importance by the rates of use of sulfa drugs today. No one would call for sulfa to replace other antibiotics. Indeed, no one would expect that all antibiotics have the same mechanism. The discovery of sulfa as an antibiotic by Gerhard Domagk in 1932 was a culmination of Ehrlich’s idea that there could be a “magic bullet” to cure a disease and it did not imply that the first magic bullet would the only magic bullet or there would be only one kind of magic bullet.

I would like to participate in the celebration of 75 years since John Cade’s discovery and divide my discussion into the following sections:

1 Introduction.
2 Were early studies of lithium’s action definitive?
3 What is the meaning of “gold standard”, “first line”, or “drug of choice”?
4 Is lithium superior to the anticonvulsant mood stabilizers valproate and carbamazepine?
5 Is lithium superior to second-generation antipsychotics as a mood stabilizer?
6 Were first-generation D-2 blockers also mood stabilizers?
7 Do we have a mechanism of action of lithium worth teaching to medical students or residents?
8 Does response to lithium define bipolar disorder and is bipolar disorder always an indication for use of lithium?
9 Lithium and Suicidality.
10 The Dangers of the Intellectual Cult Around Lithium and the Importance of an Empirical Patient-Centered Therapeutic Armamentarium

## 2. Were Early Studies of Lithium’s Action Definitive?

Cade’s first report of lithium’s usefulness in acute mania was a series of about a dozen cases. His insight into the possible utility of lithium in mania was based on theories of the pathophysiology of mania, uric acid, and the solubility of lithium urate: all speculations now known to be completely false. He had performed some small rodent studies finding a sedative effect of acute lithium injection. However, chronic oral lithium treatment in rodents is not considered to be sedative and acute peritoneal lithium as Cade gave causes painful peritoneal ulceration that certainly reduces rodent activity level but is no indication of psychoactive effect. Cade’s early discovery could therefore be considered a comedy of errors rather than an inspiring example of a rational-based discovery. His publication was certainly a brave act. It was a thrust at a paradigm change in world psychiatry which was on the cusp of emerging from psychoanalytic dominance. The story of Cade’s discovery cannot be told without the fact that he did not go on to use lithium and actually forbade the use of lithium in his hospital after the original discovery. Lithium after Cade became a major medication in the treatment of congestive heart failure by cardiologists, who after WWII had just begun to understand the role of sodium accumulation in the body as the mechanism of pulmonary edema and congestive heart failure. Lithium chloride tastes salty and was recommended by cardiologists for congestive heart failure as a replacement for sodium chloride in the diet. A wave of fatalities due to lithium intoxication spread around the world and lithium use was condemned worldwide as a sodium substitute in congestive heart failure. Lithium might have been forgotten in psychiatry as well if not for the work of Mogens Schou in Denmark and Samuel Gershon in the United States. Mogens Schou began his lithium studies perhaps based on Cade’s report but historians suggest that he may have been even more familiar with work on lithium in affective disorder performed by Danish psychiatrists in the early 1900s and published only in Danish. As often occurs in science, the technology led the way. The development of flame spectrometry allowed the easy measuring of lithium levels during lithium treatment. Schou started his work with studies in dogs and found that lithium could be administered safely if blood levels were monitored and kept within an empirically determined range. This allowed him to begin clinical trials of lithium and he published his seminal controlled clinical trial using the “mirror method” in 1970. This study, for which Schou received much highly deserved historical credit, was not accepted immediately by everyone in the field. As an example, Barry Blackwell, a distinguished psychiatrist, published a blistering critique of Mogen Schou’s study and methodology which he found unconvincing. British and American use of lithium in bipolar disorder was delayed by these critics. Sam Gershon had been a student of Cade’s in Australia and upon moving to the United States began studies of lithium with the aim of understanding its biochemical effects on the brain. He conceived the idea that lithium as a simple ion might give us unique insights into the pathophysiology of the brain in psychiatric disorder. His translational approach assumed rather than studied the actual clinical usefulness of lithium. Indeed, when the discoveries of valproate and carbamazepine as mood stabilizers occurred, several critical reviews were published claiming that lithium was of unproven effectiveness in bipolar disorder. In retrospect, the studies performed in the 1960s and 70s did not use the same rating scales as are used today, did not have the same level of blindness as used today, and did not have the large numbers of patients used in pharmaceutical research today. While lithium clearly has the longest history of use for bipolar disorder, it is hard to know how to include or exclude the early studies from meta-analyses of lithium’s efficacy.

## 3. What Is the Meaning of “Gold Standard”, “First Line”, or “Drug of Choice”?

The “gold-standard” treatment in medicine is the best available treatment at a given time. In most areas of medicine this is a very dynamic parameter and the “gold standard” of treatment changes as newer and better treatments become available. The “gold standard” certainly shouldn’t be the “latest treatment” because often as drugs lose their patented status, pharmaceutical companies synthesize “me-too” drugs that are very similar to a previous “gold standard” but with no proven additional efficacy. These “me-too” drugs are more profitable than the drug that has become generic and are highly promoted by advertising. The Food and Drug Administration (FDA) demands that such a “me-too” compound be shown to be more efficacious than placebo in at least two large clinical trials but does not demand before registration that the drug be shown to be better than the previous “gold standard”. Therefore, in the eyes of many clinicians, the latest, most expensive drug becomes the “gold standard”. This is emphatically not true. However, in other areas of medicine the word “gold standard” can sometimes be confused with the concept of the “classic drug”. For instance, digitalis could be said by some to be the “gold standard” for the treatment of congestive heart failure. It certainly was discovered very early on in the history of medicine, is somewhat unique in being a natural product found in the leaves of the foxglove plant, and has also been highly heuristic because of its specific action on the sodium pump in the heart cells which led to discovery of the ion channels and natural regulation of heart function. However, digitalis itself is rarely used nowadays and has been supplanted in clinical use by other compounds which have much wider therapeutic indices, less toxicity, and in some cases greater effectiveness. Digitalis is the classic cardiac glycoside, the classic treatment of congestive heart failure, but not the “gold standard” for every patient today with a diagnosis of congestive heart failure.

Another concept in medicine different from the concept of gold standard is the concept of a “first-line treatment”. A first-line treatment is a treatment which a physician may choose for treatment of a specific disorder in a newly diagnosed patient who has not failed in any other treatment. In most diagnoses, there are many first-line treatments. For instance, hypertension may be treated by any number of drugs with any number of different mechanisms of action: for instance, (a) a calcium-channel blocker, of which several are available; (b) an angiotensin-converting enzyme inhibitor (ACE), any number of which are available; (c) an angiotensin receptor blocker, any number of which are available; (d) a thiazide diuretic, any number of which are available; and (e) an alpha receptor antagonist, any number of which are available. All of these drugs may be used as first-line monotherapy. Very often in hypertension treatment, monotherapy is insufficiently effective and combinations of any of the “first-line treatments” represent acceptable “second-line treatment”. For cases refractory to such combinations, there are approved drugs that are given as second line, only after the failure of first-line drugs, but they have more side effect risks, more complex pharmacokinetic interactions, less favorable pharmacodynamics (such as the need to be given several times a day), or in some countries for legitimate reasons are more expensive. In many areas of medicine, such as metastatic or recurrent cancer, there are also third-line drugs that are only given to patients who have failed with first- and second-line treatment.

By contrast with the concept of “gold standard” or “first line”, the concept of “drug of choice” is less well defined. Often when doctors talk about the “drug of choice”, they might be basing their view on what might be best not for all patients with a given diagnosis but for a very specific patient with a unique combination of diagnosis, comorbid disorders, coexisting drug treatment, or economic possibilities. Clearly, the concepts of “gold standard”, “first line”, and “drug of choice” should not be confused with the concepts of (a) “what everyone is asking about”, (b) what makes me as a doctor feel good as a medical professional, (c) what I learned about in medical school, or (d) what my own area of research is.

In psychiatry, Electroconvulsive Therapy (ECT) is sometimes called the “gold standard” of antidepressant treatment. This chapter is not a review of ECT but every fair clinical scientist would agree that ECT is very rarely the “first line” of antidepressant treatment, if depression is defined in the same way as in epidemiological studies that show an incidence of about 20% of the population. ECT might be “first line” in the relatively rare disease of melancholia or acute psychotic depression. ECT, while very effective in the rare disease of melancholia in the short term, is far from a practical long term prophylactic treatment of most depressions that are recurring. It would certainly not be a “gold standard” in the treatment of a chronic resistant depression because the response rate in such patients is low, even if it might be a reasonable third- or fourth-line treatment for such patients. ECT, as almost everyone would agree, is not a “drug of choice” for most depression, even though it is on rare occasions necessary and effective and the right treatment to give, for acute and pervasive suicidality, for instance. Given these many nuances that are so important in medicine, this author has been disturbed by a recent academic crusade to call lithium “more than first line” in bipolar disorder, which sounds like gold standard [4].

Much of the use of the term “gold standard” in relation to lithium is performed by basic scientists who are trying to understand the biochemical mechanism of lithium as a simple ion on the same column of the periodic table as sodium and potassium and find it tantalizing to study in many biochemical systems (see Section 7 of this chapter). These biochemical studies, in order to introduce the reason for the study of lithium biochemistry, call lithium the “gold standard” for treatment of bipolar disorder. However, this large body of biochemical research, much of which has not been replicated and none of which has led to a clear proven mechanism of action of lithium, also cannot be taken in any sense as proof of lithium’s efficacy in bipolar disorder.

The soaring prices of gold in the international economic market after the Donald Trump administration has greatly weakened international belief in the dollar as a reserve currency, but has nothing to do with the chemistry of gold as a mineral element and everything to do with mankind’s psychological evaluation of the future of economics. The “gold standard” as a phrase teaches its own lesson: USA President Roosevelt took the dollar off the “gold standard” to a large degree in 1933 and USA President Nixon totally eliminated the connection of the dollar to gold in 1971. Both actions were economically rational, based on good economical science, and obviously successful to the patient if the patient is considered both the USA and the world economy.

Lithium is a classic treatment of bipolar disorder and is one of several first-line treatment choices available to the discerning physician based on past history of patient response, patient compliance to blood testing, patient kidney health and general health, patient side effect preference, availability of lab testing, and family history of lithium response. It is not a “gold standard”. We have come a long way in 75 years since lithium was the only option for bipolar patients [7].

## 4. Is Lithium Superior to the Anticonvulsant Mood Stabilizers Valproate and Carbamazepine?

Carbamazepine’s potential use in bipolar disorder was discovered by Okuma in Japan and published as a controlled study in Japanese in 1973 [8]. The discovery seems to have been serendipitous and the hypothesis of using carbamazepine, which was a well-known anticonvulsant for bipolar disorder, is not clear. I discussed this with Okuma himself at the 1982 conference in Munich on bipolar disorders, but the answer was not clear to me. Okuma was honored with the International College of Neuropsychopharmacology (CINP) Founders Award for his discovery of carbamazepine at the 2010 CINP congress in Hong Kong when I was president of CINP, but Okuma could not come to receive his prize and died not long afterwards. The mood-stabilizing properties of valproate were discovered by Hinderk Emrich in Germany based on reports in French and German that valproic acid and its amide derivative valnoctamine were sedative. Emrich followed up his early case studies with small controlled trials, all published in German. It may be of significance that lithium valproate and carbamazepine were all discovered by non-Americans who were willing and able to do clinical exploration in patients based on little evidence except their personal and perhaps idiosyncratic theories. All three of them are heroes in my eyes: Okuma and Emrich as well as Cade. Carbamazepine was taken up by Robert Post at the National Institutes of Health (NIH) within a theoretical framework that epilepsy and bipolar disorder may represent aspects of the same kind of recurrent abnormal brain neuronal discharge as bipolar disorder. Post’s studies are heuristic and interesting. However, few of his studies generated definitive efficacy data for carbamazepine in bipolar disorder. No pharmaceutical company was interested in a “use patent” for carbamazepine in bipolar disorder since it had already come “off-patent” for epilepsy. Therefore, the quality of research on carbamazepine’s efficacy for bipolar disorder remains limited to this day. More recently, a derivative of carbamazepine, oxcarbazepine, was patented, and a large-scale trial showed its efficacy in prophylaxis in bipolar disorder. While some clinical scientists insist that clinical trial data must relate to each specific chemical entity as requiring separate proof, my scientific epistemology is based more on the recent book by Judea Pearl, *The Book of Why: The New Science of Cause and Effect* [9], in which Pearl strongly and convincingly argues that statistics only make sense after scientific hypotheses include biological causation theory; thus, I think the success of oxcarbazepine supports the efficacy of carbamazepine in bipolar disorder. By contrast with carbamazepine, the American FDA was willing to give a special-use patent to the valproate derivative called “divalproex sodium”. Some people chuckle at this decision while others find it cynical and disturbing. Divalproex is a simple salt of valproic acid and dissolves in the stomach into valproate and is no more deserving of a special patent than lithium sulfate would be deserving of a separate set of studies comparing it to lithium carbonate. However, the FDA decision had the very positive effect of motivating the pharmaceutical company Abott to fund critical studies of valproate (“divalproex sodium”) vs. lithium vs. placebo in bipolar disorder in several phases of the disease. These classic studies were led by Charles Bowden (of blessed memory). They showed that valproate had some efficacy in some phases of bipolar disorder and lithium did also, compared to placebo, but lithium was certainly not superior to valproate.

The BALANCE study is often seen as showing superiority of lithium to valproate [10]. The numbers were 31% episode-free for valproate vs. 41% for lithium: a barely statistically significant effect and not a decisive difference in my eyes. Not much specificity here. For a particular patient 41% vs. 31% is not as decisive as the side effect profile. BALANCE did not find clinical predictors. In his FDA pivotal 12-month study of divalproex and lithium, Bowden found superiority of valproate over lithium, which was not different from placebo in most measures. These are not to the point in any case because none of these facts replicate in the Kishi et al. [11] meta-analysis and in any case the differences between valproate and lithium in the aforementioned studies are small.

The model that I support is in the figure. We have here overlapping treatments, none of which are specific overall to the illness we call bipolar disorder. Sometimes a patient responds to one and sometimes to another.

## 5. Is Lithium Superior to Second-Generation Antipsychotics as a Mood Stabilizer?

**Figure 1:**
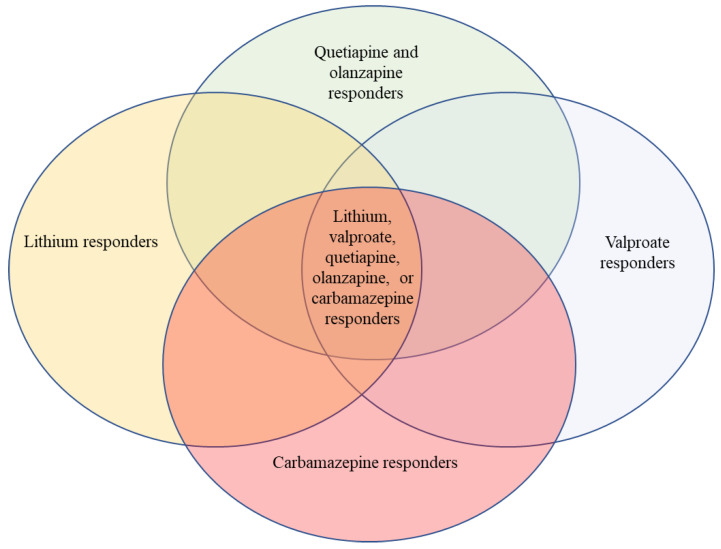
The above figure illustrates roughly the fact that some people respond to lithium, another group responds to valproate, another group responds to carbamazepine, and another group responds to some dopamine blockers; many patients with bipolar disorder respond to any of these four classic mood stabilizer groups, and some respond to only one or two. There is good clinical data to call all of these treatments mood stabilizers, but each is distinct chemically and biochemically. Printed with modifications and permission from Wolters Kluwer; Belmaker: “*Lithium*” in Kaplan and Sadock’s *Comprehensive Textbook of Psychiatry*, edition 11, 2024 [14].

When the second-generation antipsychotics became available, it was logical and obvious to try them in mania, since first-generation antipsychotics had been universally known to be highly effective in mania. Many studies had shown that the first-generation antipsychotics reduced activity and aggressiveness more rapidly than lithium in mania but others indicated that lithium in mania had greater patient acceptability because the patient felt that his mood improved more naturally and more gradually compared to the rapid straightjacket effect of first-generation antipsychotic treatment. Second-generation antipsychotics beginning with olanzapine seemed to reduce acute mania more like lithium clinically: there were few extra pyramidal side effects (EPS) and no sense of akinesia or thought emptying. There was more sedation than lithium, which proved a significant clinical advantage in acute mania. Few clinicians were prepared, however, for the fact that olanzapine and other second-generation antipsychotics turned out to be prophylactic in both phases of bipolar disorder [12]. The pharmaceutical industry, while its motives may be by definition financial, should be commended for not adhering to the boundaries of the *Diagnostic and Statistical Manual of Mental Disorders* (DSM) and for aggressively pursuing a program of extending by large clinical trials the indications of olanzapine and other second-generation antipsychotics into the realm of affective disorders. Olanzapine was found to be equally effective to lithium in the prophylaxis of mania and depression [13]. Even more surprisingly, olanzapine and particularly quietiapine, (Seroquel) were found to be equally effective to SSRIs in the treatment of acute bipolar depression as well as prophylaxis of bipolar depression. These new second-generation antipsychotics have numerous biochemical properties, many of which differ between the different second-generation antipsychotics. Most block the 5HT2 receptor as well as the D4 receptor but others such as amisulpride do not. Recent partial agonists/antagonists such as aripiprazole have little activity at the 5HT2 receptor but seem to be highly effective in the prophylaxis of bipolar disorder both in manic and depressive phase. It has not yet been determined whether second-generation antipsychotics that are D-2/5HT2 blockers are more efficacious in a different subgroup of bipolar patients than the partial agonists such as aripiprazole or even the D3 dominant partial agonists such as cariprazine or brexpiprazole. The DSM-5 emphasis on bipolar disorder as an entity encourages treatment trials with entry criteria of bipolar disorder and endpoints of results of the rating scale, but subgroup analysis becomes post hoc and statistically suspect. The DSM-5 concept of bipolar disorder has become so dominant that clinical intuition about possible subtypes that could then be tested with specific drugs has been effectively suppressed. It would be baffling if these very varied biochemical receptor acting drugs all worked in the same way on the heterogenous clinical syndrome of bipolar disorder (Figure 1).

## 6. Were First-Generation D-2 Blockers Also Mood Stabilizers?

Some current clinicians are unaware that first-generation antipsychotics were probably mood stabilizers. See Bond et al., “Depot antipsychotic medications in bipolar disorder: a review of the literature” [15] or Littlejohn and Cookson: “Depot antipsychotics in the prophylaxis of bipolar affective disorder” [16]. Authors who have worked outside of the US and Europe know that antipsychotics are used by huge populations all around the world effectively for the treatment of bipolar disorder [17]. I have in my files a piece of correspondence about this issue, which I wrote 30 years ago to many leaders in the bipolar field. The responses that I have in my files, which have also been preserved in the CINP/American College of Neuropsychopharmacology (ACNP) historical archives of the history of psychopharmacology by Tom Ban, showed that no leader in the bipolar field in that time denied the efficacy of first-generation antipsychotics in bipolar disorder, including prophylaxis. They merely answered that the risk of tardive dyskinesia was too high to recommend (then atypical) antipsychotics as opposed to lithium. Moreover, historians of psychopharmacology have clearly pointed out the powerful influence of the intellectual needs of psychopharmacologists themselves in defining the nature of mood stabilizer vs. antipsychotic vs. antidepressant. As an emerging field, we wanted to find clinical/biochemical specificity. This historical pressure could be the subject of a whole article but I urge the reviewer to think about it. I cannot think of one drug that has dopamine receptor blocking properties, from clozapine to olanzapine to aripiprazole to cariprazine to lurasidone, that has not been shown to be an effective treatment for one or both poles of bipolar disorder without exacerbation of the other pole—the definition of a mood stabilizer, as I have read.

For some, the issue of tardive dyskinesia ends first-generation antipsychotics. This statement bothers me greatly in 2025. The second-generation antipsychotics have led to an epidemic of diabetes and obesity in bipolar patients and other patients that will be on our conscience for decades. The fact that first-generation antipsychotics cause tardive dyskinesia must be weighed against the terrible clinical side effects of second-generation antipsychotics in each patient specifically. I have written about it in my book *Psychopharmacology Reconsidered: A Concise Text Exploring the Limits of Diagnosis and Treatment* [18].

## 7. Do We Have a Mechanism of Action of Lithium Worth Teaching to Medical Students or Residents?

Thirty-five years ago, I co-authored an editorial [19] attempting to adjudicate between the two leading theories of that era: adenylate cyclase inhibition via G-proteins, and inositol monophosphatase (IMPase) inhibition. Both theories seemed promising based on the biochemical evidence available then. Neither has yielded the therapeutic insights or new treatments that we hoped for. This pattern—of initially exciting findings that fail to translate into clinical advances—has repeated itself across multiple generations of lithium research.

### 7.1. The Generational Pattern of Lithium Research

As I discussed in detail in a 2004 New England Journal of Medicine article [20], lithium research has exhibited a remarkable pattern: in each era of psychopharmacology, lithium has been found to affect whatever biochemical system was at the frontier of investigation. When monoamine metabolites dominated research in the 1960s and 70s, lithium was found to affect norepinephrine metabolism. When receptor studies emerged in the 1970s and 80s, lithium prevented dopamine receptor supersensitivity. When second messenger systems became central in the 1980s and 90s, lithium inhibited key enzymes in multiple messenger pathways. When gene expression studies dominated in the late 1990s and 2000s, lithium affected transcription factors. Most recently, when neurogenesis and neuroprotection became prominent, lithium was found to affect Bcl-2 and cell survival pathways.

This generational shifting might reflect lithium’s genuine complexity as a simple ion affecting fundamental cellular processes. Alternatively, it might reveal more about the sociology of scientific research than about lithium’s true mechanism of action. Each generation of researchers has found what they were equipped and incentivized to look for.

### 7.2. Key Biochemical Findings: A Critical Summary

Rather than exhaustively reviewing the biochemical literature—which I have performed comprehensively elsewhere [21]—I will critically evaluate the major mechanistic theories that have shaped lithium research and teaching.

The Cyclic AMP Hypothesis: Our laboratory demonstrated in the 1980s that lithium at therapeutic doses completely blocked the cyclic AMP response to epinephrine in euthymic bipolar patients [21], replicating in humans what had been found in animal systems. We developed receptor binding assays showing that lithium could inhibit G-protein activation in rat brain tissue [22]. However, this effect proved highly dependent on magnesium concentrations in the experimental medium, and its relevance to human therapeutic conditions remains unclear. More critically, if cyclic AMP inhibition were lithium’s primary mechanism, we should have been able to develop clinically useful compounds that more specifically target this pathway. We have not.

The Inositol Depletion Hypothesis: Perhaps the most influential theory proposed that lithium’s inhibition of inositol monophosphatase at therapeutic concentrations (Ki = 0.8 mM) depletes cellular inositol, particularly in overactive neurons, thereby providing specificity to lithium’s action [23,24,25]. This elegant “inositol depletion” theory dominated lithium research for two decades. Our laboratory extensively studied this mechanism, demonstrating that inositol administration could stereospecifically reverse certain behavioral effects of lithium in rodents [26]. We developed knockout mouse models where inositol monophosphatase-1 knockout mice showed lithium-like behaviors [27].

However, this theory has significant problems. Most critically, when ebselen—a compound that inhibits inositol monophosphatase and lowers brain inositol—became available for testing, it failed to replicate lithium’s effects in key behavioral models [28]. This negative finding, published by our laboratory in 2020, undermines decades of research built on the assumption that inositol depletion is the key to lithium’s clinical effects. Additionally, inositol did not function as an “antidote” for lithium toxicity as the theory would predict [29], and several studies failed to demonstrate that lithium consistently reduces brain PIP2 concentrations in primates.

The GSK-3 Hypothesis: Glycogen synthase kinase-3 (GSK-3) emerged as an attractive target when lithium was found to directly inhibit this ubiquitous kinase at concentrations near the therapeutic range (2.1 ± 0.6 mM) (41). GSK-3 plays critical roles in multiple cellular processes including metabolism, gene expression, and apoptosis [30,31]. The finding that valproic acid also inhibits GSK-3 [32] initially strengthened the hypothesis by suggesting a common mechanism for mood stabilizers. Moreover, lithium’s effects on development and Wnt signaling could be explained through GSK-3 inhibition [33,34].

However, the GSK-3 hypothesis faces the same translational problem as earlier theories: the inhibition occurs at the top of lithium’s therapeutic range or higher [35], raising questions about whether this is a mechanism of therapeutic action or toxicity. Despite considerable investment, synthetic GSK-3 inhibitors have not been successfully developed as brain-penetrable, brain-selective compounds suitable for clinical testing as lithium alternatives. The failure to translate this biochemical finding into therapeutic application after more than two decades suggests fundamental limitations in our understanding.

Other Proposed Mechanisms: Lithium’s effects on serotonin autoreceptors [36,37,38], on neuroprotective proteins like Bcl-2 [39,40,41,42,43], on PAP phosphatase [44,45,46,47,48], and on glutamate systems [49,50,51,52,53] have all generated research programs and publications. Each finding is replicable within specific experimental conditions. None has led to new treatments or to a consensus understanding of lithium’s clinical action.

### 7.3. The Alzheimer’s Controversy: A Cautionary Tale

The recent enthusiasm for lithium in Alzheimer’s disease prevention illustrates the dangers of over-interpreting biochemical findings. Based on sophisticated animal studies showing GSK-3β effects on tau phosphorylation, researchers proposed that lithium might prevent amyloid deposition and delay Alzheimer’s disease [54,55]. Press releases and media coverage were extensive. Many Alzheimer’s patients and their families were encouraged to begin lithium treatment.

However, the lithium concentrations used in these studies were often 1000 times lower than any concentration ever shown to inhibit GSK-3 in biochemical assays—a fact that should have induced immediate skepticism. Claiming that a microdose (1/1000th of the usual therapeutic dose) of lithium can prevent Alzheimer’s is as scientifically defensible as claiming that 1/1000th of the usual LSD dose prevents depression. The comparison to microdosing psychedelics is intentional: both claims reflect wishful thinking rather than rigorous science.

Well-controlled clinical trials of lithium in Alzheimer’s disease have been predominantly negative. Yet, this failed hypothesis has been recycled to support broader claims about lithium as a “neuroprotective agent,” creating an undeserved halo effect that obscures rather than illuminates lithium’s actual clinical utility. It is especially troublesome that these researchers touted lithium orotate as a special compound. Li in medicine is a salt that dissolves in the stomach into Li^+^ ions. Only Li is measured in blood. Orotate is metabolized.

### 7.4. Modern Approaches: Gene Expression and Stem Cell Studies

Recent research has taken hypothesis-free approaches using large datasets. Akkouh and colleagues [56] studied lithium’s effects on thousands of genes simultaneously using gene chips, finding that lithium increases some mRNA levels while decreasing others. This is analogous to observing that lithium affects an orchestra’s performance: you can see that some wind instruments become faster, some percussion becomes slower, and some violins change pitch—but this does not tell you what effect lithium is having on the conductor himself. To develop a new lithium-like drug, we would need to identify the proximal mechanism that causes these hundreds of simultaneous gene expression changes [57].

Stern and colleagues have pioneered the use of stem cells differentiated into neurons from skin biopsies of bipolar patients versus controls, as well as immortalized lymphocytes from both groups [58]. This innovative approach allows study of human tissue without invasive procedures and addresses whether lithium has different effects in bipolar lithium-responsive patients than in controls or non-responders. Complex effects on cell membrane excitability have been found, though which effects are relevant or specific remains unclear.

### 7.5. Why No Mechanism After 70 Years?

Several explanations warrant consideration:

Lithium may have no single proximal mechanism. As a simple ion affecting fundamental cellular processes involving sodium, potassium, and magnesium, lithium might exert its clinical effects through multiple, redundant pathways. This “many mechanisms” hypothesis contradicts Occam’s razor but may reflect biological reality.

Bipolar disorder itself may be heterogeneous. If bipolar disorder as currently diagnosed represents multiple distinct pathophysiologies, then searching for a unifying mechanism of lithium action is misguided. Lithium may work through different mechanisms in different patient subgroups—an explanation that fits clinical observations better than the “one disease, one mechanism” model.

The therapeutic concentration is the problem. Unlike drugs that bind to specific receptors at nanomolar concentrations, lithium works at millimolar concentrations where it affects numerous systems simultaneously. This lack of specificity at the molecular level may make it impossible to identify a primary mechanism distinct from secondary effects.

Our experimental systems may be inadequate. Most biochemical studies use artificial in vitro systems or simplified animal models that may not capture the relevant clinical biology. The repeated failure to translate promising animal findings into human therapeutics suggests fundamental limitations in our research paradigms.

### 7.6. What Should We Teach Medical Students?

Given this state of affairs, what is worth teaching to medical students and residents about lithium’s mechanism of action?

What we should NOT teach: We should not present any single mechanism as “the answer” to how lithium works. Teaching that lithium works primarily through inositol depletion, or GSK-3 inhibition, or any other single pathway, misrepresents the current state of knowledge and sets students up for confusion when they encounter contradictory information later.

What we SHOULD teach:

1. Honesty about uncertainty: Lithium’s mechanism of action is unknown despite seven decades of intensive research. This is not a knowledge gap that will be filled by one more study—it represents a fundamental challenge in psychiatric neuroscience.
2. The pattern of research: Lithium has been found to affect virtually every biochemical system investigated at multiple levels, from ion channels to gene expression. This may reflect genuine biological complexity rather than inadequate research.
3. The contrast with other psychotropics: Unlike antipsychotics (dopamine D-2 blockade) or antidepressants (monoamine reuptake inhibition), we lack even a simplified textbook-level explanation of lithium’s action that bears a defensible relationship to truth.
4. Clinical implications: The lack of a known mechanism means we have no laboratory test to predict lithium response, no rational approach to designing improved lithium-like compounds, and no way to identify which biochemical abnormality in bipolar disorder lithium is correcting.
5. The lesson for psychiatry: Lithium’s story teaches humility. Effective treatments can exist without mechanistic understanding. Clinical efficacy and biochemical mechanism are separate questions. The assumption that understanding mechanism is “the key to understanding” a psychiatric disorder may itself be flawed.

### 7.7. Implications for the “Gold Standard” Debate

The absence of a known mechanism of action has important implications for lithium’s status in treatment guidelines. Those who argue that lithium is the “gold standard” for bipolar disorder sometimes invoke its long research history and the extensive investigation of its biochemical effects as supporting evidence for its superiority. However, as this section demonstrates, 70 years of biochemical research has not revealed a mechanism of action, has not led to improved lithium-like compounds, and has not provided methods to predict which patients will respond.

The mystique surrounding lithium—as a simple ion that should be easy to understand, as a naturally occurring element with hypothesized specificity for bipolar disorder—has arguably hindered rather than helped clinical practice. It has encouraged an ideological attachment to lithium that sometimes overrides individualized clinical judgment about which treatment is best for a particular patient.

Basic scientists studying lithium biochemistry often begin their papers by declaring lithium “the gold standard for bipolar disorder” as justification for their research. But this large body of biochemical research, much of which has not been replicated and none of which has led to a proven mechanism of action, cannot be taken as proof of lithium’s clinical superiority over other mood stabilizers. The lack of pharmaceutical company support for lithium research means that comparative studies with adequate statistical power have been limited. We simply do not know whether lithium’s mood-stabilizing effects are more potent, more durable, or more specific than those of valproate, carbamazepine, or second-generation antipsychotics.

Summary: After 70 years of research, we must acknowledge that lithium is a clinically useful compound whose mechanism of action remains unknown. This is not a temporary gap in knowledge but may reflect fundamental complexity in both lithium’s biology and bipolar disorder’s pathophysiology. Rather than continuing to invest in mechanistic studies that fail to translate into clinical advances, we might better serve patients by focusing on comparative effectiveness research, prediction of individual treatment response, and development of truly novel therapeutic approaches based on emerging understanding of mood disorder neurobiology.

## 8. Does Response to Lithium Define Bipolar Disorder and Is Bipolar Disorder Always an Indication for Use of Lithium?

We do not know which cases of bipolar disorder will respond to lithium. Martin Alda and Paul Grof are two talented and wonderful researchers whom I respect greatly. I have gone over their papers for the umpteenth time and they find correlations that explain a tiny percentage of the variation in lithium response that can be explained by the clinical presentation or genetics. For instance, “Genetic variance with response to lithium treatment in bipolar disorder: a genome-wide association study” in *The Lancet* [59] studied 2563 patients collected by 22 participating sites from the International Consortium on Lithium Genetics and found a single locus associated with response to lithium monotherapy. Given that all data on lithium therapy in bipolar disorder finds no single Mendelian pattern, it is highly unlikely that this single locus explains a significant part of the variance [60]. Another paper by Paul Grof entitled “Sixty Years of Lithium Responders” [61] states in the abstract, “Despite promising reports, particularly from molecular genetics, we are still waiting for a biological elucidation of the stabilizing effect of lithium. The most useful predictor of lithium stabilization to date has been the patients’ clinical profile based on a comprehensive clinical assessment; complete remission and other characteristics of episode clinical course, low psychiatric comorbidity…”. This seems like the definition of a patient who would respond to other treatments as well to lithium: “the good prognosis patient”. The paper presents no evidence that these phenomena predict lithium responders rather than just “responders”. Grof goes on to say that the clinical picture of the lithium responders approximates the classical Kraepelinian description of a manic-depressive patient. NO, NO, NO! Kraepelin’s definition of manic-depressive illness included patients with highly psychotic manias and highly psychotic depressions. These patients were clearly excluded from Grof’s definition of good lithium responders but were included in the NIMH groups by Goodwin and others, showing that lithium response is not dependent on the presence or absence of severe psychosis in the mania and also many papers showing the excellent response of many schizoaffective patients to lithium [62]. Many clinicians seem to confuse two different concepts: Statement #1: Lithium works better in classical bipolar 1 patients than in other forms of psychiatric disorder. Statement #2: Lithium works better than valproate, carbamazepine, olanzapine, or quetiapine in classical bipolar 1 patients.

Statement #1 has some evidence from the Alda and Grof work, but contradicts much work of Goodwin at NIMH, such as Carlson and Goodwin’s “The Stages of Mania” 1973 [62], where the presence of psychosis in mania does not predict lithium response. Statement #2 has not really been tested to my knowledge but it should be realized that classical bipolar 1 according to Grof and Alda is not Kraepelinian manic-depressive disorder nor is it a genetic subgroup of bipolar disorder. It is merely mild bipolar disorder with many good prognostic features. Perhaps statement #1 would be equally true of carbamazepine, valproate, and olanzapine in the current climate where bipolar 2 disorder is more frequent than bipolar 1 disorder and many borderline patients are diagnosed as bipolar. Without comparing the lithium efficacy to that of valproate and dopamine blockers, this whole area of research has gone down a rabbit hole.

Some say that Kishi et al. [11,63] contradict other meta-analyses demonstrating lithium’s superiority over placebo. These scholars are incorrect. They are answering the wrong question. No one has ever claimed in recent decades that lithium is not better than placebo. The point studied by Kishi et al. [11,63] is whether it is better than compounds with completely different mechanisms of action such as the antiepileptics and the dopamine receptor blockers.

Some say that it is critical to compare data quality rather than efficacy. There is some sense in which this idea is correct: any compound that has drug company backing will most likely have large short-term studies. Lithium data, however, has much smaller sample sizes and has rarely been studied with modern multicentered designs. I think that this is comparing apples and oranges and I stand behind my statement, “Lithium is only one of many effective treatments for bipolar disorder”. We do not have a football game-style design as to whether I am for or against lithium. I am not against lithium and agree that the mechanism of many, if not all, psychiatric treatments is unknown. Lithium is not uniquely unfathomable, but it is not a mystically heuristic holy grail either. The point is that lithium was claimed over these last 75 years to be such a simple ion that we would soon understand it. This has not turned out to be the case and it is time to face up to that fact. I do not agree that such dramatic effects as lithium produces are rarely seen in treatments with other mood stabilizers. I have seen bipolar patients respond wonderfully over many years to carbamazepine, to valproate, to olanzapine, and yes, to 1 mg of haloperidol.

## 9. Lithium and Suicidality

A large number of epidemiological studies support the concept that patients on lithium have fewer serious suicide attempts and fewer successful suicides. In and of itself, this finding bears only tenuous relationship to lithium’s effects in bipolar disorder. True, bipolar disorder carries a high risk of suicide. But so do schizophrenia, unipolar disorder, and borderline personality disorder. Lithium’s effect on suicidality may be an entirely independent effect similar to the hypothesized effect of ivermectin in COVID, although ivermectin was originally purposed as an antiparasitic drug. On the other hand, an epiphenomenon may be at work here. The only other drug reported in large epidemiological studies to have an anti-suicidal effect is clozapine. Clozapine has almost no biochemical similarities to lithium. The only similarity is that both clozapine and lithium require frequent blood monitoring. Perhaps the effects of lithium on suicidality are merely an epiphenomenon of the increased patient surveillance required by the lithium blood testing. Many such associations exist in the medical literature and I find it an unconvincing fact when it is used in support of lithium’s uniqueness.

## 10. The Dangers of the Intellectual Cult Around Lithium and the Importance of an Empirical Patient-Centered Therapeutic Armamentarium

A new book about critical psychiatry by Awais Aftab (*Conversations in Critical Psychiatry, Oxford University Press*, ref. [64]) discussed the importance of science’s ability to self-criticize and the particular importance of this for psychiatry. Admirably, he has emphasized that critics also need to be self-critical. There is no question that an “antipsychiatry movement” exists that denies the existence of mental illness, specific diagnoses, or the possibility that chemical treatments would benefit individuals with mental disorders. This antipsychiatry movement is not self-critical and may not be possible to engage in a beneficial fashion. However, not all critiques of psychiatry and psychopharmacology are tainted with ideological antipsychiatry. The writings of Montcrief [65,66] have pointed out that antidepressants may be blunting all emotion rather than specifically lifting abnormal depressed mood. This hypothesis is far from proven but worthy of serious consideration in psychopharmacology. Robin Murray [67] and equally serious psychopharmacological scholars also question whether dopamine-blocking drugs are truly antipsychotic or whether their therapeutic benefit derives from restricting human imagination and creative thinking and thereby including subjective impairments that are a serious cost for the patient under treatment. The possibility that antidepressant and antipsychotic treatments are working by helping some patients but inducing a cost in others leads to a therapeutic calculus that is very different than an approach based on the idea that psychopharmacology treats specific diseases with specific treatments. For some critics of psychopharmacology, lithium has remained as an exception, a rare example of a treatment which has been assumed because of its biochemical specificity to be truly related to the pathophysiology of bipolar disorder. I do not think that this has been proved. The need for studies of the effects of lithium on the personality of the bipolar patient who benefits from it is great. Even in those patients where the benefit greatly outweighs the psychological or medical costs and side effects, there is no reason to discount in advance the complaints of many patients that lithium restricts their range of affective expression or creativity. All medical and psychiatric treatment is a balance between hoped-for benefits and appropriately considered side effects of any external intervention. Lithium should not be seen as an exception because the rule of this cost/benefit analysis is the very basis of medicine and psychiatry.

I still use lithium in the clinic and find it very beneficial in many patients with bipolar disorder, some with recurrent unipolar disorder and many with schizoaffective disorders; atypical antipsychotics are often but not always equally useful and are very convenient [68]. Given the long period of lithium usage in many of my patients, kidney effects including progression to renal dialysis and transplant have become a very real issue [69]. Basic research on the mechanism of lithium action has not led to application of Occam’s razor, but clinical usage surely justifies the adage that there is no free lunch.

This review is a response to the exuberance of the Mahli and Bauer editorial [4]. Such exuberance is in such contrast to the practice seen by residents in psychiatry everywhere that it leads to confusion and undermines the legitimacy of academic leadership. I agree that a drug’s specificity is not directly related to its efficacy. In the case of lithium, the history of psychopharmacology connected the two and this is the reason that I address the two together. Lithium’s use as a gold standard has generated thousands of articles using lithium in the test tube presenting results as possible mechanisms of bipolar disorder. My claim is that such biochemical studies should use lithium, an antiepileptic, and a dopamine blocker in each study if any inferences about the underlying illness are to be made. I think the history of this field has indeed been damaged by the idea that lithium is “the gold standard”.

Lithium can play a treatment role in a variety of conditions. Some praise the clinical experience of lithium in the context of bipolar disorder over the “long term”. I do not know of any long-term controlled studies of lithium vs. placebo. All long-term studies are of selected patients followed based on various clinical and genetic phenotypes. I do not agree that lithium has the largest database. In fact, lithium just 25 years ago was a subject of controversy as to whether it worked at all until lithium was used as a control treatment in several large pharmaceutical company-sponsored trials of other compounds. Most of the lithium database in the long term uses small sample sizes in highly selected patient groups.

Long-term good lithium response could equally well be a predictor of natural course of illness and would be equally valid with any other medication if tried [70]. The original, most gripping description of bipolar disorder, *A Mind that Found Itself* by Clifford Beers in 1908 [71], was of a patient who had five years of continuous hospitalization with severe bipolar mood alterations that ended with complete recovery and a lifetime of productive social activities, long before the introduction of lithium.

## Data Availability

No new data were created or analyzed in this study. Data sharing is not applicable to this article.
